# Antegrade Wire Tracking of the Retrograde Tract (ATRT): A Novel Technique for Percutaneous Coronary Intervention for Chronic Total Occlusion

**DOI:** 10.7759/cureus.65148

**Published:** 2024-07-22

**Authors:** Asit Das, Gaurav Lakhani, Tusharkanti Patra, Santosh Kumar

**Affiliations:** 1 Department of Cardiology, Institute of Post Graduate Medical Education and Research and Seth Sukhlal Karnani Memorial Hospital, Kolkata, IND; 2 Department of Periodontology and Implantology, Karnavati School of Dentistry, Karnavati University, Gandhinagar, IND

**Keywords:** primary percutaneous coronary intervention (pci), atrt, retrograde approach, antegrade approach, cto: chronic total occlusion

## Abstract

Background: Chronic total occlusion (CTO) lesions are the most challenging subset of coronary lesions. For lesions with a failed antegrade approach, the initial retrograde, followed by a combined retrograde and antegrade approach, remains the mainstay of therapy.

Objective: The study evaluated a technique of initial retrograde followed by an antegrade approach to treat lesions with a failed antegrade approach.

Methods: We have adopted this technique to treat 31 CTO lesions with a failed antegrade approach, where a floppy wire was advanced antegrade through the tract created by a retrograde balloon advanced over the retrograde wire (antegrade wire tracking of the retrograde tract (ATRT)), which was advanced into the aorta retrogradely.

Result: In 31 patients with failed antegrade approaches, the ATRT technique was tried, which was successful in 25 patients (the success rate was 80.6%). There was a failure to cross the microchannel in four patients, although angiographically, it looked promising. In two patients, it was impossible to advance the microcatheter or the smallest profile balloon retrogradely until the entire length of the CTO body. So, a reverse controlled antegrade and retrograde subintimal tracking (CART) was performed on these two patients excluded from the study.

Conclusion: ATRT is a useful technique for CTO percutaneous coronary intervention (PCI) for patients with failed antegrade approaches with acceptable success rates. The procedure is safe in terms of procedural complications.

## Introduction

Chronic total occlusion (CTO) lesions represent the most complex and challenging subset of coronary artery blockages that interventional cardiologists face in their clinical practice [1]. These blockages are characterized by complete plaque obstruction of blood flow through a coronary artery for a prolonged period, typically over three months [2]. Treating CTO lesions requires specialized techniques and expertise due to their intricate anatomy, often involving extensive calcification, tortuosity, and bridging collaterals [3].

The antegrade approach, where devices and wires are advanced through the normal blood flow within the artery, is generally the preferred initial strategy for CTO percutaneous coronary intervention (PCI) [3]. PCI is a minimally invasive procedure aimed at restoring blood flow in blocked arteries [4]. However, certain specific CTO scenarios necessitate a primary retrograde approach [5]. This approach involves accessing the blocked artery from the opposite direction, utilizing collateral vessels to reach the occlusion site.

Ostial CTO lesions, affecting the very beginning of the coronary artery, along with lesions exhibiting an unclear proximal cap (the starting point of the blockage) and extensive calcification, are prime candidates for a primary retrograde approach [6]. The complexity of these lesions often renders the antegrade approach less feasible or successful [7]. Furthermore, when the initial antegrade approach fails to achieve revascularization [8], a combined retrograde and antegrade approach becomes the standard treatment strategy. This hybrid approach leverages the strengths of both techniques to navigate and ultimately recanalize the occluded artery.

Among the various techniques employed in CTO PCI, controlled antegrade and retrograde subintimal tracking (CART) and reverse CART have gained significant popularity [9]. These techniques involve creating a pathway within the artery wall's layers to bypass the blockage and re-establish blood flow [10]. While these established techniques have proven valuable, our study explored a novel approach termed antegrade wire tracking of the retrograde tract (ATRT).

ATRT is a unique technique that combines elements of both antegrade and retrograde approaches [11]. In this approach, after establishing a retrograde path using a wire and balloon, a floppy wire is advanced antegrade through the newly created tract [10]. The wire is made of stainless steel wire, usually 316L, aged 630, and has a shape memory alloy of nitinol or CoCrMo with high yield strength. The balloons are made up of polyethylene terephthalate (PET), nylon, polyethylene with additives, polyvinyl chloride (PVC), and polyurethane used along with nylon. This floppy wire is guided by the retrograde wire, which is advanced into the antegrade guiding catheter (a tube used to access the coronary arteries) or the aorta (the main artery leaving the heart). ATRT offers a promising alternative, particularly in patients with failed antegrade attempts who possess usable collateral vessels.

Our study sought to evaluate the feasibility, safety, and efficacy of the ATRT technique in a specific patient population. By meticulously documenting procedural details and outcomes, we aimed to contribute valuable insights to the evolving field of CTO PCI. The potential benefits of ATRT, including its ability to overcome challenges encountered in traditional approaches, could significantly impact the treatment landscape for patients with complex CTO lesions.

It is important to note that ATRT, like any interventional procedure, carries inherent risks and requires meticulous execution. Thorough pre-procedural planning, careful patient selection, and a comprehensive understanding of the technique's nuances are essential to ensure optimal outcomes. By further refining and optimizing ATRT, we can enhance our ability to tackle the most daunting coronary blockages and improve the quality of life for countless individuals suffering from CTO-related cardiovascular disease.

## Materials and methods

The study was conducted at the Institute of Post Graduate Medical Education and Research outpatient department at Seth Sukhlal Karnani Memorial Hospital, Kolkata, India, from July 1, 2022, to September 30, 2023. It was conducted according to the Declaration of Helsinki. The institutional review board reviewed and approved the study on January 4, 2022 (IPGM/IRB/2021/234). The sample size (n) was calculated using the formula: n = 2(Za)2 C SD2/d2, where SD = anticipated standard deviation from the previous studies = 8; Za = value of Z when a = 5% =1.76; d = minimum expected mean difference = 3. Therefore, the minimum sample size was calculated to be 28 subjects. Considering few dropouts, 31 patients were recruited to perform a combined antegrade and retrograde approach with the ATRT technique and presented with a failed antegrade approach for a coronary CTO lesion. Strict adherence to "nil by mouth" instructions was given to all the patients before surgery. All patients signed written informed consent, agreeing to undergo a combined antegrade and retrograde approach.

**Figure 1:**
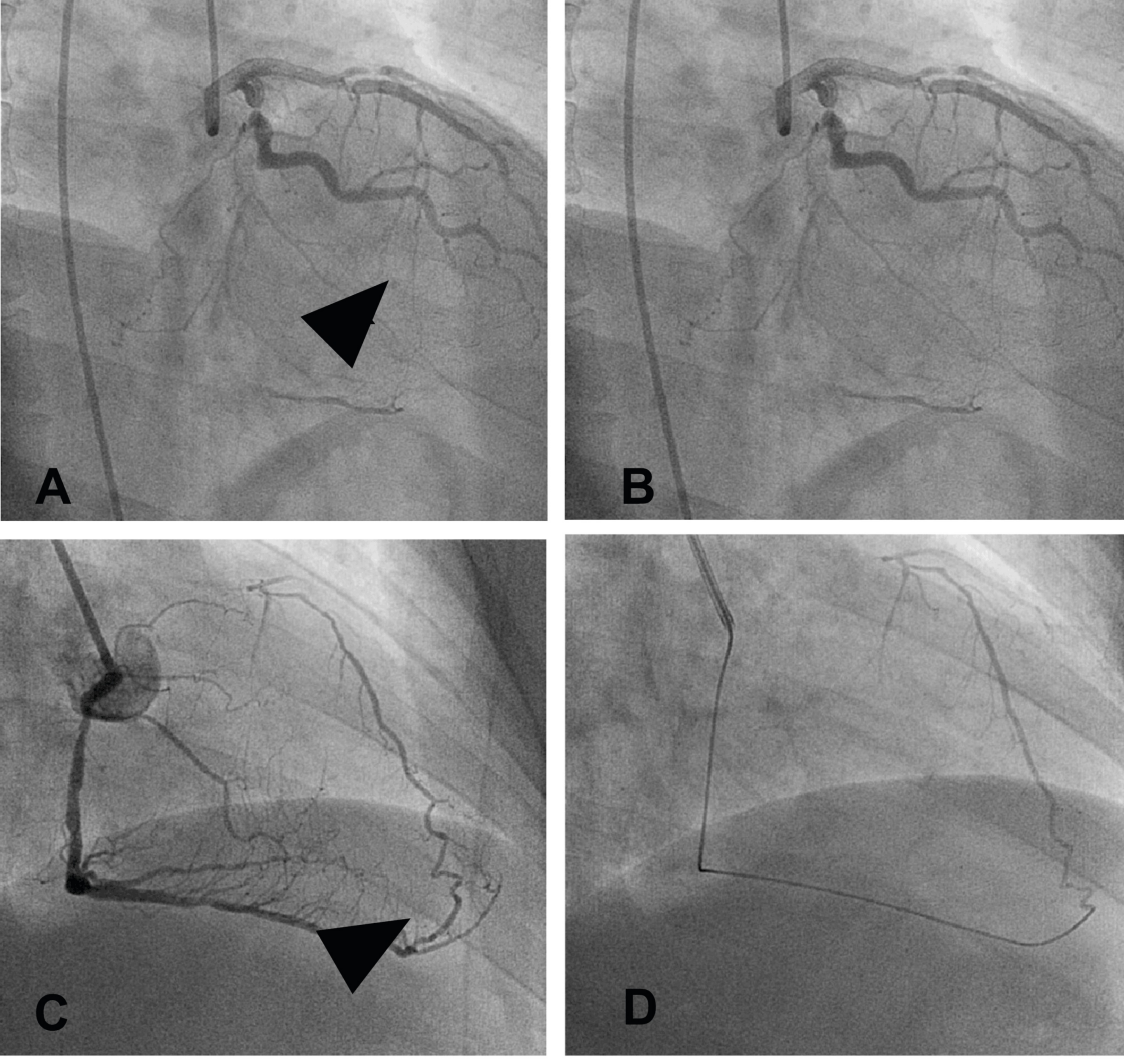
Successful collaterals: (A) septal collateral (arrowhead) from LAD supplying RCA PDA; (B) selective angiogram of the channel through the microcatheter; (C) epicardial collateral (arrowhead) from the PDA branch supplying LAD; and (D) selective angiogram of the same channel through the microcatheter. Photo credit: Asit Das LAD = left anterior descending artery; PDA = posterior descending artery; RCA = right coronary artery

**Figure 2:**
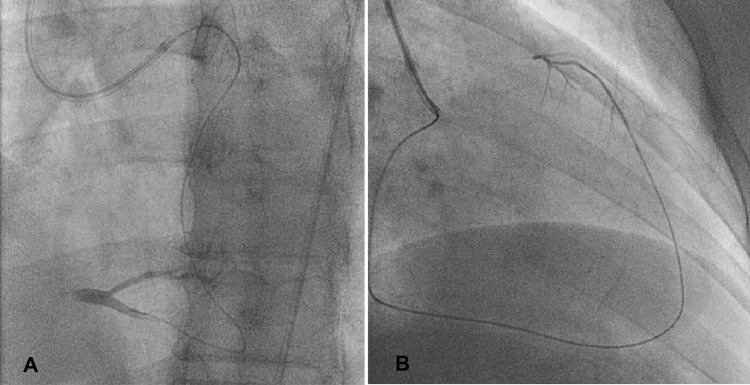
(A) After crossing the collateral injection through the microcatheter, the distal RCA bifurcation and the distal cap were visible in the same patient as in Figure 1A. (B) The microcatheter tip injection showed the distal CTO cap in the LAD (the same patient as in Figure 1B). Photo credit: Asit Das RCA = right coronary artery; CTO = chronic total occlusion; LAD = left anterior descending artery

Initially, two 6-F arterial accesses (both radials or one radial and one femoral) were taken. Injection unfractionated heparin was administered at a dose of 100 U/kg, and after one hour, 1000 U was repeated every 30 minutes. A 90-cm guiding catheter (GC) was used to engage the donor artery for the retrograde approach, and a 100-cm GC was used to engage the target artery. A simultaneous injection through both the GCs was taken in two orthogonal views. The target lesion was assessed for its morphology (proximal cap tapered or abrupt cutoff, presence of a side branch at the proximal cap), approximate length by the quantitative coronary angiography (QCA) method, and fluoroscopically visible calcium. An approximate CTO length of <20 mm was considered short, ≥20 mm and <40 mm were termed long, and ≥40 mm was considered very long [12]. Calcification was considered severe when radiopacities were noted without cardiac motion before contrast injection and involved both sides of the arterial wall, termed as moderate when radiopacities involved one side of the arterial wall, and mild when scattered radiopacities were noted along the CTO body [13]. The most suitable retrograde channel (either septal or epicardial) was identified (Figures 1, 2).

**Figure 3:**
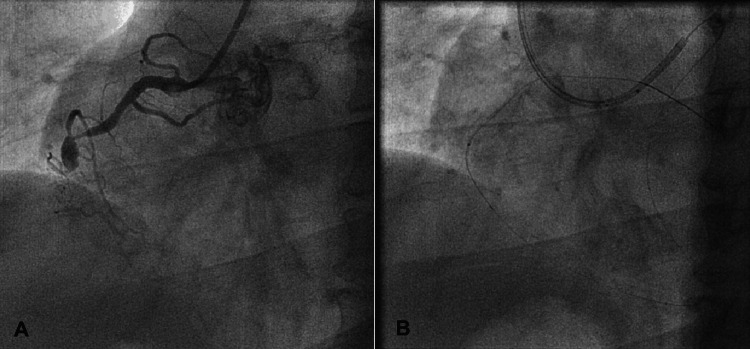
The retrograde wire is hanging in the aorta (A) mid-RCA CTO lesion; (B) the retrograde wire has crossed the lesion and is hanging in the aorta, along with a small balloon (SC 1.25 × 10 mm) dilatation of the CTO body. Photo credit: Asit Das CTO = chronic total occlusion; RCA = right coronary artery; SC = semi-compliant

The second most suitable retrograde channel was also looked for in all cases. A 150 cm microcatheter was taken (Corsair Pro XS (Asahi Intecc, Gurugram, India) or Finecross (Terumo, Gurugram, India), according to availability) to engage the retrograde channel through the retrograde GC. A new Suoh 03 guide wire (Asahi Intecc) was used to cross septal collaterals in all cases. The Fielder XTR (Asahi Intecc) guide wire was tried in failed cases. The Fielder XT guide wire crossed epicardial collaterals. In case of failure, a new Suoh 03 wire was used. After crossing the microchannel to the target vessel, a tip injection through the microcatheter was performed in all cases to visualize the distal cap (Figure 3).

**Figure 4:**
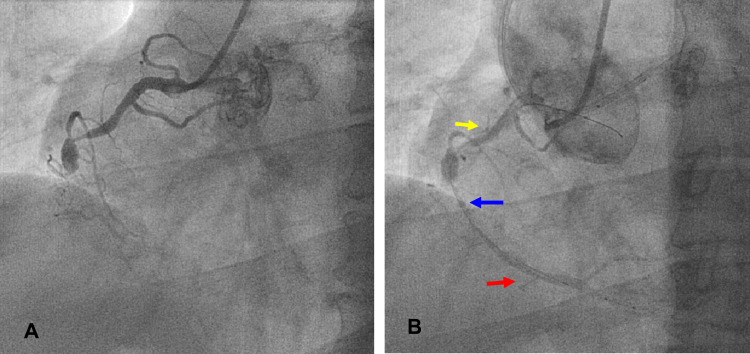
The retrograde tract connects the proximal and distal true lumen (A) mid-RCA CTO lesion; (B) the retrograde tract (blue arrow) connects the distal true lumen (red arrow) and the proximal true lumen (yellow arrow). Photo credit: Asit Das CTO = chronic total occlusion; RCA = right coronary artery

To cross the lesion retrogradely, the Gladius Ex14 (Asahi Intecc) guide wire (a hydrophilic, non-tapered tip wire with a 3gm tip load) was our first choice. Because of its hydrophilic nature, it has a higher possibility of tracking microchannel retrogradely. With a higher tip load compared to contemporary hydrophilic wires, it has a higher penetrating force. In case of failure, Gaia 2 (Asahi Intecc) guide wire was used. The retrograde wire was tried to advance into the antegrade GC. In cases of failure, snaring was attempted through the antegrade GC. If snaring failed, the retrograde guidewire was left hanging in the aorta (Figure 4).

**Figure 5:**
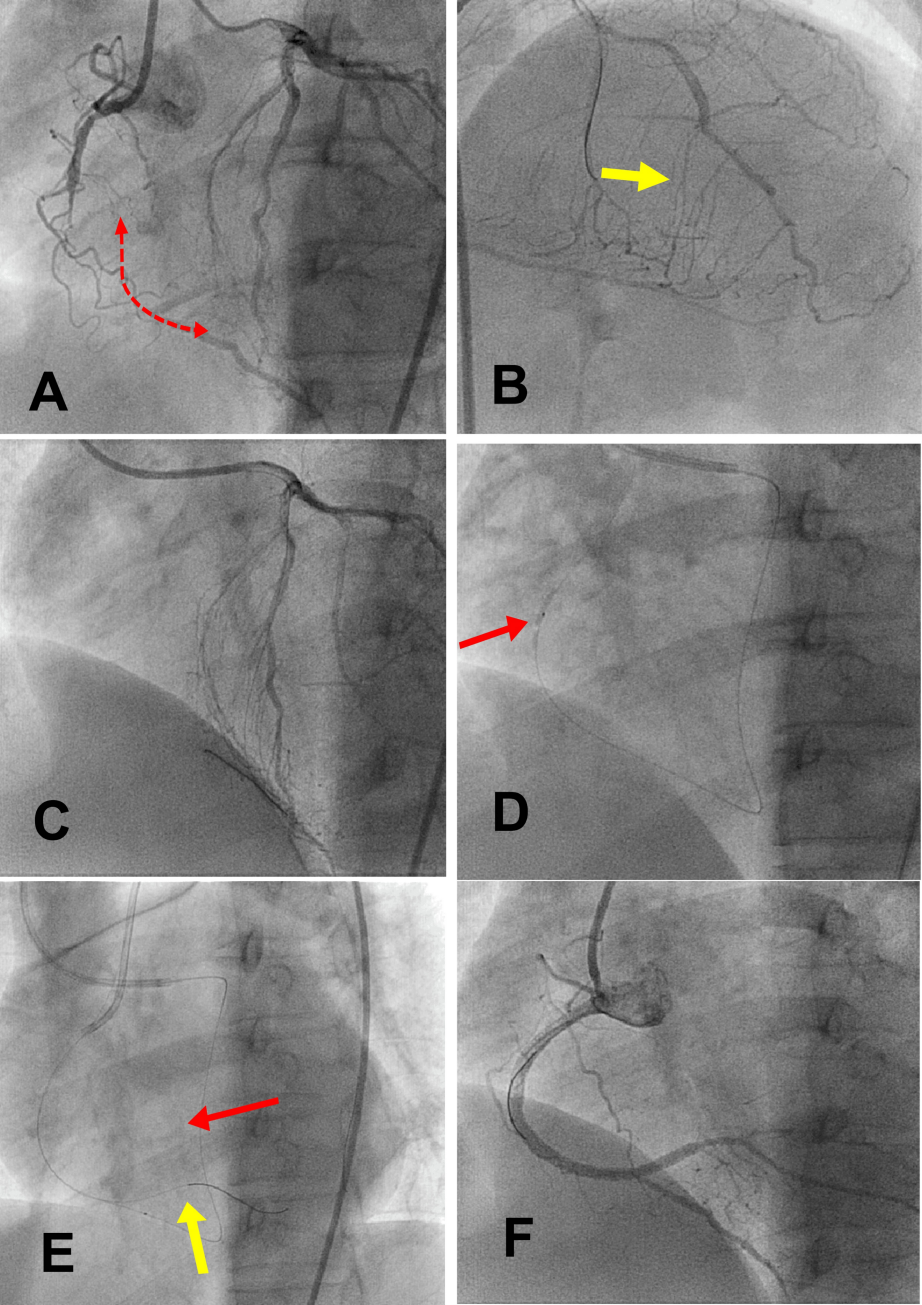
ATRT: a step-by-step method for an RCA CTO: (A) simultaneous injection into both coronaries to estimate the length of CTO (red dotted arrow), proximal and distal cap location. (B) Identification of the best usable collateral. S3 was the best channel in this case (yellow arrow). Note the floppy wire location at S1. (C) A new Suoh 03 retrograde wire in the RCA PDA. (D) Small (1.25 x 15 mm) SC balloon dilatation of the retrograde tract (red arrow). (E) The antegrade wire (yellow arrow) tracked the retrograde tract and positioned at the PLV branch, while the retrograde wire with its balloon (red arrow) in position. (F) Final result after stenting the lesion. Photo credit: Asit Das ATRT = antegrade wire tracking of the retrograde tract; CTO = chronic total occlusion; PDA = posterior descending artery; PLV = posterior left ventricular; RC = right coronary artery; SC = semi-compliant

The microcatheter was slowly advanced over the retrograde wire to the proximal true lumen. After a few "to and fro" movements, it was pulled to the distal true lumen. Then, the whole retrograde tract was dilated with a monorail semi-compliant small balloon with nominal pressure. This created a “retrograde tract” that connected the proximal and distal true lumens in the target vessel (Figure 5).

A floppy wire (Runthrough floppy, Terumo) was used from antegrade GC to track the retrograde tract and reach the distal true lumen. After confirmation of the antegrade guide wire in the distal true lumen, the retrograde wire and the dilating balloon were withdrawn. Retrograde GC was injected to exclude injury to the used collateral channel. Bed preparation followed by stent deployment was performed over the antegrade guide wire. Adequate post-dilatation with non-compliant balloons was performed in all cases. The procedure was abandoned if the antegrade wire positioning was not achieved with either a fluoroscopic time of 60 minutes, a procedure time of 90 minutes, or both, or a contrast volume exceeding 3 mL/kg of body weight. Procedural success was defined as restoration of antegrade flow, with a TIMI (thrombolysis in myocardial infarction) grade 3 flow with final residual stenosis of ≤30% determined by QCA [14]. Specific attention was given to early detection of any developing complication (donor artery ischemia, septal injury, cardiac tamponade). In-hospital major adverse cardiac events (MACE) were defined as death, non-Q and Q-wave myocardial infarction, or the need for target vessel revascularization [14].

## Results

Of the 31 patients, the ATRT technique was successful in 25 patients (the success rate was 80.6% (25/31)). In four (12.9%) (three septal, one epicardial) patients, this method failed to cross the microchannel, although angiographically, it looked promising. In two patients, it was impossible to advance the microcatheter or the smallest profile balloon retrogradely until the full length of the CTO body. Therefore, reverse CART was performed on these two patients, who were excluded from the study. The demographic profile of these 25 patients is summarized in Table 1.

**Table 1:** Demographic profiles of the patients ACS = acute coronary syndrome; CSA = chronic stable angina; CABG = coronary artery bypass graft; CAD = coronary artery disease; LVEF = left ventricular ejection fraction; n (%) = number and percentage; SD = standard deviation

Profiles of the Patients		Variables	Numerical Data
Sociodemographic profile	Age	In years ± SD	64.5 ± 10.5
	Sex	Male n (%)	18 (58%)
		Female n (%)	13 (42%)
Medical history		Diabetes n (%)	10 (40)
		Hypertension n (%)	15 (60)
		Dyslipidemia n (%)	15 (60)
		Prior ACS n (%)	10 (40)
		CSA n (%)	15 (60)
		Stent failure n (%)	5 (20)
		Prior CABG n (%)	2 (8)
		Triple vessel CAD n (%)	10 (40)
		Double vessel CAD n (%)	10 (40)
		Single vessel CAD n (%)	5 (20)
		LVEF n (%) ± SD	48±12
Social habit/addiction		Smoker n (%)	15 (60)

Of these 25 patients, 15 were men, and 10 were women (3:2). The mean age was 64.5 years (± 10.5 years). The youngest patient was 51 years old, and the oldest patient was 76 years of age. A total of 10 (40%) patients were diabetic, 15 (60%) were smokers, 15 (60%) were hypertensive, and 15 (60%) were hyperlipidemic. Additionally, 10 (40%) patients had a history of acute coronary syndrome at least one year back. No patient had any history of acute events in the last year of their presentation. Fifteen (60%) patients had presented with chronic stable angina refractory to optimum medical therapy. Five (20%) patients had a history of stent failure. Two (8%) patients had prior CABG. There were 10 (40%) patients who had triple vessel coronary artery disease (CAD), 10 (40%) had double vessel CAD, and five (20%) patients had single vessel CAD. The baseline mean left ventricular ejection fraction was 48% (±12%). Table 2 provides important baseline information on the lesion characteristics.

**Table 2:** Details of the lesion characteristics RCA = right coronary artery; LAD = left anterior descending coronary artery; LCX = left circumflex coronary artery; n (%) = number and percentage

Variables	Types	Numerical Data
Target vessel	RCA n (%)	16 (64)
	LAD n (%)	8 (32)
	LCX n (%)	1 (4)
Lesion length (mm)	<20 n (%)	8 (32)
	≥20-<40 n (%)	15 (60)
	≥40 n (%)	2 (8)
Lesion calcification	No n (%)	12 (48)
	Mild n (%)	7 (28)
	Moderate n (%)	5 (20)
	Heavy n (%)	1 (4)
Bridging collaterals (%)	n (%)	22 (88)
Proximal cap	Tapered n (%)	8 (32)
	Abrupt cutoff n (%)	12 (48)
	Presence of side branch n (%)	5 (20)

The right coronary artery was the target vessel in 16 (64%) patients. The left anterior descending artery was the target vessel in eight (32%), and the left circumflex was the target vessel in one (4%) patient. The lesion length was short in eight (32%) patients, long in 15 (60%) patients, and very long in two (8%) patients. No fluoroscopic evidence of calcium in the CTO body existed in 12 (48%) patients. Whereas seven (28%) patients showed mild, five (20%) moderate, and one (4%) showed heavy calcification in the CTO body. A total of 22 (88%) lesions showed bridging collateral at the CTO site. The lesions at its proximal end were tapered in eight (32%), 12 (48%) abrupt cutoff, and five (20%) side branches. The procedural characteristics are shown in Table 3.

**Table 3:** Procedural characteristics. n (%) = number and percentage; atm pr = atmospheric pressure; RT = run-through; SD = standard deviation

Variables	Types	Numerical Data
Successful collaterals	Septal	18 (72)
	Epicardial	7 (28)
Wires used to cross the collaterals	New Suoh 03 (septal) n (%)	15/18 (83.3)
	Fielder XTR (septal) n (%)	3/18 (16.66)
	Fielder XT (epicardial) n (%)	-
	New Suoh 03 (epicardial) n (%)	-
Wire for retrograde crossing	Gladius Ex 14 n (%)	18 (72)
	Gaia 2 n (%)	7 (28)
Preparation of retrograde tract	Micro catheter only n (%)	5/25 (20)
	1.25 × 10 mm Tazuna balloon n (%)	12/20 (60)
	1.50 × 10 mm Tazuna balloon n (%)	8/20 (40)
	Inflation pressure of balloon (atm pr) ± SD	11 ±3
Antegrade wire	RT floppy (%)	25/25 (100)

Of these 25 patients, the septal channel was used successfully in 18 (72%) patients and epicardial collateral in seven (28%) patients. Of these 18 patients with successful septal collateral crossing, a new Suoh 03 guidewire was used in 15 (83.3%). However, in three (16.7%) patients, a new Suoh 03 could not cross the septal channel, which was crossed with Fielder XTR. Of the seven patients who successfully used epicardial collateral, Fielder XT was successful in four (57%) patients, and a new Suoh 03 as the second wire was successful in three (43%) patients. Corsair Pro XS was used in 15 (60%) patients, and in 10 (40%) patients, the Finecross microcatheter was preferred according to availability. In no case was the microcatheter changed. The retrograde crossing was with Gladius EX 14 guidewire in 18 (72%) patients, and in seven (28%), it was upgraded to Gaia 2 guidewire. The usable “retrograde tract” was prepared with a 1.25 mm semi-compliant balloon in 17 (68%) (Tazuna 1.25 × 10 mm, Terumo) patients and with a 1.5 mm semi-compliant balloon in eight (32%) (Tazuna 1.5 × 10 mm, Terumo) patients. The mean inflation pressure of the balloons was 12.5 (±3.1) atmospheric pressure. Antegrade guidewire was run through floppy (Terumo) in all cases. The mean diameter of balloons used for antegrade bed preparation was 2.75 (±1.3) mm. Non-compliant balloons have a mean inflation pressure of 18.0 (±2.5) atmospheric pressure. The mean fluoroscopic time was 30.6 minutes (±10.3), and the total procedure time was 62.8 (±16.6) minutes. There was no collateral injury and no cardiac tamponade. Two (8%) patients had transient donor artery ischemia, which responded to an intracoronary nitroglycerine injection of 100 µg. There was no MACE in our hospital study.

## Discussion

Up to 20% of patients who undergo diagnostic coronary angiograms have reported having one or more coronary CTO (occlusion of more than one month) lesions [1]. Successful CTO PCI results in relief of angina, which translates to improved physical function and quality of life [9]. It also causes improved LV function, a reduction of ischemia, and complete revascularization, ostensibly for improved survival in the absence of symptoms [15-17]. Once a patient has been identified as an appropriate candidate for intervention on a coronary CTO, the next step is to consider strategies for crossing the occlusion to perform PCI. The primary antegrade approach is the preferred strategy in most cases with the presence of a tapered, unambiguous proximal cap [18]. Antegrade wire escalation is preferred in lesions with unambiguous proximal caps [19]. In cases of failure or progress of the wire into the subintimal space, the parallel wire technique or intravascular ultrasound (IVUS)-guided reentry is the next step one should follow [20]. An ambiguous proximal cap with a side branch at the proximal cap favors primary IVUS-guided wiring. Antegrade dissection reentry is another important technique for antegrade CTO PCI [21]. A primary retrograde approach is required to treat an ostial CTO lesion, a lesion with an ambiguous proximal cap and a very long and calcified CTO body [22].

However, for lesions with a failed antegrade approach, initial retrograde followed by a combined retrograde and antegrade approach remains the mainstay of therapy. CART and reverse CART are the most preferred techniques; other techniques include antegrade wire escalation, parallel wire technique, antegrade dissection reentry, retrograde wire escalation, and septal and epicardial collateral channel tracking. In the CART technique, a balloon was retrogradely inserted into the subintimal space within the CTO lesion and dilated to make the target space for the antegrade wire access [22]. This space was connected to the distal true lumen so that the antegrade guidewire could easily reach the distal true lumen. On the contrary, in reverse CART, the balloon was inserted into the subintimal space within the CTO lesion antegradely and dilated to make the target space for retrograde wire penetration [14].

Our technique is similar to the CART technique, with subtle differences. In this technique, a small, low-profile monorail balloon was inserted over the retrograde wire until the antegrade proximal true lumen and dilated the whole course of the wire (true lumen/subintimal space/partial true lumen and partial subintimal) with nominal pressure to create a tract that connects both the proximal and distal true lumen. A floppy wire was now used antegradely to track the retrograde tract created by the balloon to reach the distal true lumen. In CART, the antegrade wire was always stiffer (with a higher tip load) to enter the subintimal space created by the retrograde balloon. But, in our technique, a floppy wire was always used antegradely. The retrograde balloon used in our technique (maximum diameter up to 1.5 mm) was much smaller than the CART technique (1.5-3.0 mm). The inflation pressure in our study was 12.5±3.1 atmospheric pressure, in contrast to the CART technique, which was 6-18 atmospheric pressure. Like the CART technique, there was no collateral injury in our study. Our procedure had an acceptable success rate of 80% [10,23,24].

Limitations of the study

Our present study was limited by its relatively small sample size. Moreover, this was a single-center, single-operator experience. Our study assessed the feasibility, safety, and immediate success rate of this technique. Therefore, it is possible that the success rate and complication rate may differ in a larger patient-size series. The feasibility of this technique needs to be tested in patients with very long CTO lengths and dense calcification. So, a large randomized multicenter study with a longer follow-up period was required.

## Conclusions

ATRT is a valuable technique in CTO PCI when the initial antegrade approach fails. It involves a hybrid strategy, beginning with a retrograde approach to cross the occlusion and then transitioning to an antegrade approach for reentry into the true lumen distal to the occlusion. This combined approach has shown promising results, with success rates comparable to other CTO PCI techniques. While ATRT may initially seem complex, studies have demonstrated its safety profile, with procedural complications and in-hospital MACE occurring at rates similar to or lower than those seen in other CTO PCI strategies.
